# The caudate nucleus undergoes dramatic and unique transcriptional changes in human prodromal Huntington’s disease brain

**DOI:** 10.1186/s12920-019-0581-9

**Published:** 2019-10-16

**Authors:** Filisia Agus, Diego Crespo, Richard H. Myers, Adam Labadorf

**Affiliations:** 1 Bioinformatics Nexus, Boston University, Boston, 02118 USA; 2 Bioinformatics Program, Boston University, Boston, 02118 USA; 3 Department of Neurology, Boston University School of Medicine, Boston, 02118 USA

**Keywords:** Huntington’s disease, Neurodegeneration, Bioinformatics, High throughput mRNA sequencing, Differential expression, Machine learning

## Abstract

**Background:**

The mechanisms underlying neurodegeneration in the striatum of Huntingon’s Disease (HD) brain are currently unknown. While the striatum is massively degenerated in symptomatic individuals, which makes cellular characterization difficult, it is largely intact in asymptomatic HD gene positive (HD+) individuals. Unfortunately, as striatal tissue samples from HD+ individuals are exceedingly rare, recent focus has been on the Brodmann Area 9 (BA9), a relatively unaffected region, as a surrogate tissue. In this study, we analyze gene expression in caudate nucleus (CAU) from two HD+ individuals and compare the results with healthy and symptomatic HD brains.

**Methods:**

High-throughput mRNA sequencing (mRNA-Seq) datasets were generated from post-mortem CAU of 2 asymptomatic HD+ individuals and compared with 26 HD and 56 neurologically normal controls. Datasets were analyzed using a custom bioinformatic analysis pipeline to identify and interpret differentially expressed (DE) genes. Results were compared to publicly available brain mRNA-Seq datasets from the Genotype-Tissue Expression (GTEx) project. The analysis employed current state of the art bioinformatics tools and tailored statistical and machine learning methods.

**Results:**

The transcriptional profiles in HD+ CAU and HD BA9 samples are highly similar. Differentially expressed (DE) genes related to the heat shock response, particularly HSPA6 and HSPA1A, are common between regions. The most perturbed pathways show extensive agreement when comparing disease with control. A random forest classifier predicts that the two HD+ CAU samples strongly resemble HD BA9 and not control BA9. Nonetheless, when genes were prioritized by their specificity to HD+ CAU, pathways spanning many biological processes emerge. Comparison of HD+ BA9 with HD BA9 identified NPAS4 and REST1/2 as potential early responders to disease and reflect the active disease process.

**Conclusions:**

The caudate nucleus in HD brain is dramatically affected prior to symptom onset. Gene expression patterns observed in the HD BA9 are also present in the CAU, suggesting a common response to disease. Substantial caudate-specific differences implicate many different biological pathways including metabolism, protein folding, inflammation, and neurogenic processes. While these results are at best trends due to small sample sizes, these results nonetheless provide the most detailed insight to date into the primary HD disease process.

## Background

Huntington’s Disease (HD) is a devastating neurodegenerative disease caused by an expanded trinucleotide CAG repeat in the HTT gene. The striatum, comprising the caudate nucleus (CAU) and putamen, is the primary affected brain region in HD where as many as 90% of neurons are lost in late stage disease. Although other brain regions, such as the cerebellum and cerebral cortex show the hallmarks of HTT protein intranuclear inclusions, they are relatively free of neurodegeneration [1, 2]. While studying the striatum directly in post mortem HD brains is preferable, the lack of neurons in these highly degenerated tissues makes interpretation difficult. CAU samples from post-mortem human brains of asymptomatic HD gene positive (HD+) individuals, who died before evidence of significant degeneration has occurred, avoid this difficulty but are extremely rare.

Previously, we performed unbiased transcriptomic analysis with high throughput sequencing (mRNA-Seq) in pre-frontal cortex Brodmannn Area 9 (BA9) of twenty HD and forty-nine non-neurological control brain samples[3]. Neuroinflammation and developmental pathways were implicated by the differentially expressed (DE) genes from this study, and there was evidence that every major resident brain cell type (i.e. both neurons and glia) is implicated in HD pathogenesis. However, since all of these individuals were symptomatic and at an advanced stage of disease at the time of death, it was unclear which aspects of the gene expression signature were causes and which were consequences of disease. Examining gene expression from brain tissue of asymptomatic HD+ individuals provides an opportunity to address this key question, as gene expression changes that are present prior to evidence of symptoms and neurodegeneration offer an opportunity to gain insight into initiating disease processes. Furthermore, comparing gene expression changes in BA9 and CAU of the same individuals enables examination of how the changes in a relatively unaffected tissue (BA9) reflect those observed in the primarily affected brain region (CAU).

The Myers lab has obtained brain tissue from BA9 of three asymptomatic HD+ individuals, as well as CAU from two of these same individuals from the McLean Brain Tissue Resource Center (BTRC). These tissues and age and sex matched controls were subjected to mRNA sequencing to assess genome wide alterations in gene expression. The HD+ expression dataset was then compared with our previous HD mRNA-Seq datasets [4], as well as BA9 and CAU mRNA-Seq samples from the Genotype-Tissue Expression (GTEx) database. The goals of this study were to 1) identify DE genes in the CAU prior to clinical onset and neurodegeneration, 2) compare DE genes between BA9 and CAU in HD+ individuals to identify region-specific and common expression patterns, and 3) identify genes involved in the early vs late disease process.

## Methods

### Human subjects

The individuals in this study are exempt as defined by the Boston University School of Medicine Institutional Review Board, due to the fact that all analyses were conducted on postmortem brain tissue.

### Differential expression contrasts

**Fig. 1:**
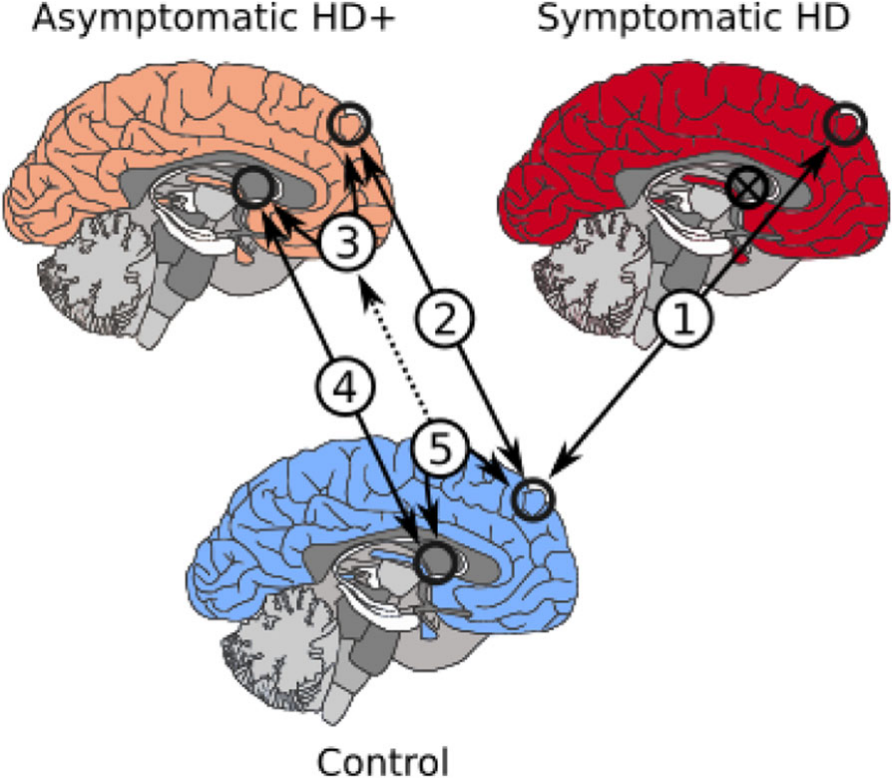
Brain region contrasts performed

Five different pair-wise contrasts were performed as described in Fig. 1 and Table 1.

**Table 1:** Sample sizes for contrasts performed

Analysis	Group A	Group B	Sample size	# DE genes	Genes detected
(1)	HD BA9	C BA9	26 vs 56	7789	32490
(2)	HD+ BA9	C BA9	3 vs 9	229	35843
(3)	HD+ CAU	HD+ BA9	2 vs 3	1199	34084
(4)	HD+ CAU	C CAU	2 vs 2	74	35554
(5)	GTEx BA9	GTEx CAU	90 vs 102	23778	34081

First number corresponds to number of samples for column sample type, e.g. for analysis (1) there were 26 HD BA9 and 56 C BA9. The number of DE genes reported have FDR <0.05. *BA9 control samples that matched the age at death were chosen from the whole control set for this analysis

This manuscript refers to specific analyses by the corresponding numbers in Fig. 1. Analysis (1) compares BA9 for symptomatic HD and neurologically normal controls. Analysis (2) compares HD+ BA9 with C BA9, identifying DE genes likely implicated in the early disease process. Analysis (3) compares HD+ BA9 with HD+ CAU, identifying DE genes caused either by disease or due to differing brain region. Analysis (4) compares HD+ CAU with C CAU, identifying DE genes implicated by the active HD disease process. Analysis (5) compares GTEx BA9 with GTEx CAU, identifying DE genes caused by difference in brain region, to assist in identifying DE genes identified in analysis (3) that are not simply a consequence of different brain region.

### Sample processing

26 symptomatic HD and 49 control BA9 mRNA-Seq libraries were used as previously described [4], and three additional control BA9 samples age-matched for the HD+ BA9 samples presented here were generated. Paired BA9 and CAU tissues from two asymptomatic HD gene positive individuals, one additional asymptomatic HD gene positive BA9 sample, and two CAU samples from neurologically normal controls were extracted and processed to generate mRNA-Seq libraries following the procedure previously described [4]. Table 2 contains a summary of the datasets used in this study, and statistics for the new samples reported in this study are found in Table 3.

**Table 2:** Sample sizes for each class

Sample type	Num samples	PMI	RIN	Age of death
HD BA9	26	16.04 ±7.65	7.29 ±0.89	59.77 ±10.42
HD+ BA9	3	24.17 ±8.63	7.9 ±0.62	51.33 ±33.56
C BA9	56	15.23 ±9.44	7.94 ±0.64	67.88 ±16.97
GTEx BA9	90	14.13 ±4.16	7.25 ±0.87	58.63 ±8.8
HD+ CAU	2	27.97 ±7.91	7.35 ±0.49	67.5 ±26.16
C CAU	2	31.24 ±9.64	7.8 ±1.27	66.5 ±21.92
GTEx CAU	102	14.03 ±4.13	7.66 ±0.76	60.07 ±6.71

PMI and Death columns are means followed by standard deviation. Complete sample information is included in Additional file 5. HD+ = asymptomatic HD gene positive, HD = symptomatic HD, C = non-neurological control, GTEx = the Genotype-Tissue Expression database, BA9=Brodmann area 9, CAU=Caudate nucleus

**Table 3:** Sample statistics

Sample ID	Status	BA9	CAU	PMI	Age of Death	Sex	CAG
H_1105	HD+	X	X	33.56	49.0	M	41
H_1104	HD+	X	X	22.37	86.0	F	41
H_1106	HD+	X		16.58	19.0	M	55
C_0113	Control		X	38.06	51.0	M	NA
C_0114	Control		X	24.42	82.0	F	NA

HD+ are asymptomatic gene positive individuals. Two HD+ individuals had both BA9 and CAU brain tissues available for analysis. Full sample statistics are included in Additional file 5

### Quality control and mRNA abundance estimation

mRNA-Seq libraries were subject to quality control and analysis using a custom pipeline. All sequencing libraries were quality- and adapter-trimmed with trimmomatic [5], and then assessed to be of high quality using fastqc [6] and MultiQC [7]. Trimmed reads were analyzed with salmon [8] to obtain mRNA abundance estimates using the GENCODE v26 gene annotation [9]. Abundance estimates from all samples were concatenated into a single matrix and normalized with the DESeq2 normalization method [10]. The normalized expression matrix was investigated for outlier samples using PCA, where no outliers were found (Additional file 2). Due to the different numbers of samples, genes in each analysis were filtered using different strategies. For analyses 1, 2 and 5, genes with more than 50% zero counts within each group was filtered out. For analyses 3 and 4 genes with more than 2 zeros and genes with more than 4 zeros was filtered out, respectively. Therefore, the genes detected for each analysis were different as seen in Table 1.

### GTEx Analysis of BA9 vs CAU

Post mortem human brain samples from BA9 and CAU brains available from the GTEx project [11] were downloaded and processed as above. After processing the samples through the quality control pipeline described above, 56 samples were removed due to differences in per base Sequence Content, over representation of sequences, or discrepancies in read length, leaving a total of 90 BA9 samples, 102 CAU samples for analysis. These 192 samples were used to form the basis of a contrast between HD+ BA9 and HD+ CAU samples.

### Differential expression analysis

Five differential expression contrasts were conducted in this study as described in the analysis matrix of Table 1 and Fig. 1. Differential expression statistics for all five analyses were assessed using DESeq2 [10], modeling counts as a function of either disease status or brain region, adjusting for age at death and sex. Differentially expressed genes were considered significant if they had FDR <0.05.

### Gene set enrichment

Gene set enrichment analysis for all DE gene lists was performed using the fgsea [12] R package in bioconductor [13] and MSigDB C2 Canonical Pathway database version 6.2 [14, 15]. GSEA statistics were calculated using each gene list sorted by descending log2 fold change, and significance was assessed for gene sets at FDR <0.05.

### Random forest predictive model to classify HD+

A random forest of decision trees was used to classify the HD+ CAU and BA9 samples as either HD BA9 or C BA9. A decision tree is a predictive model that iteratively bifurcates a set of labeled samples by identifying features (e.g. genes) that have predictive power when partitioning samples by a fixed threshold. The decision tree algorithm is a machine learning technique that is used to identify features and their levels that best partition a sample set according to given labels. For example, if gene A is expressed between 10 and 20 in one set of samples and between 30 and 40 in another, samples with an expression value less than 25 are likely to belong to one class, while samples with an expression value greater than 25 will belong to the other. If a single gene cannot perfectly divide samples into their labels, additional genes are chosen in a hierarchical fashion until samples with different labels are perfectly partitioned. Once a decision tree has been trained, it may be used to classify previously unobserved samples into the labels used in training.

Individual decision trees trained with all samples are often over-fit, so a randomization technique called random forests are used with decision trees to identify robust predictive features. Random forests perform bootstrap sampling on samples and random selection of features to build a large number of decision trees, where each is a different predictive model with different sets of features. After training, unobserved samples are applied to each decision tree in the forest and the predicted label of each is recorded and reported. The agreement of predicted labels across all trees in the forest is an indication of the predictive power of the overall dataset. A random forest where all trees classify a new sample to have the same label indicates a perfect classification. A random forest predicting a sample to be of either class with equal frequency has no predictive power.

We trained a random forest of decision trees using the HD BA9 vs C BA9 normalized counts matrix to arrive at a predictive model of genes that well classify the samples. The random forest was trained using cross-validation, where the samples are divided into training and test sets. Decision trees in the forest are built using the training samples and their predictive accuracy is assessed on the test set. In this way, cross validation enables assessing the robustness of a classifier and avoids over-fit predictive models. Each random forest contained 20,000 trees, 250 genes, 75% training sets were created with ratios of HD and Control samples which mirrored ratios present in the dataset. Prediction accuracy was assesses as the mean true positive predictions divided by the number of trees across all samples in the test set. 1000 random forests were trained in this way, and the average and standard deviation of true positive rates were recorded for each. See Table 4 for statistics on cross validation prediction accuracy.

**Table 4:** Accuracy results

	Top 250 DE	Random 250	Null
Train accuracy	1 ±0	1 ±0	1 ±0
Test accuracy	0.969 ±0.052	0.781 ±0.117	0.489 ±0.138
Sensitivity	0.971 ±0.07	0.779 ±0.175	0.49 ±0.211
Specificity	0.967 ±0.081	0.786 ±0.173	0.494 ±0.211
False positive	0.029 ±0.087	0.221 ±0.175	0.51 ±0.211
False negative	0.0033 ±0.081	0.214 ±0.173	0.506 ±0.211

Random forest cross validation prediction accuracy statistics for the HD vs C samples. The Random 250 random forests were generated by selecting from a random subset of 250 genes from the overall dataset. The Null random forests were generated by shuffling sample labels and choosing 250 genes at random

HD+ CAU and HD+ BA9 samples were applied to the random forests trained above. First, random forests built with the top 250 DE genes from (1) ranked by significance were used to predict the HD+ samples as either HD or C. We then built random forests with 250 randomly selected genes from (1), irrespective of significance, and performed classification of the HD+ samples. Finally, we built random forests with permuted sample labels and randomly selected genes to assess the basal predictive power under a null dataset. The results of these randomized random forest classifiers is included in Table 8.

### t-statistic analysis of DESeq2 log fold changes

We sought to identify genes that show different response between HD+ CAU and HD+ BA9 (3 vs 5) and early response genes from HD+ BA9 vs HD BA9 (1 vs 2). Although an interaction model estimating the difference in effect size between BA9 and CAU by HD status is a more conventional approach, the small number of samples in the HD+ group (i.e. 2 HD+ CAU vs 3 HD+ BA9) prohibits reliable fitting in this case. Therefore, we utilized a *t*-statistic methodology to quantify the difference between DESeq2 log2 fold change estimates while taking the uncertainty those estimates into account. DESeq2 implements a negative binomial generalized linear model, whose estimated coefficients are normally distributed. DESeq2 also reports the standard error of its log2 fold change estimates, enabling the calculation of a *t*-statistic corresponding to the confidence-adjusted difference in log2 fold change. Specifically, we calculate a *t*-statistic assuming both unequal sample sizes and unequal variance:

$$t = \frac{X_{1} - X_{2}}{s_{\Delta}} $$

where

$$s_{\Delta} = \sqrt{\frac{s_{1}^{2}}{n_{1}} + \frac{s_{2}^{2}}{n_{2}}}. $$

Here, *X*_1_ and *X*_2_ are the log2 fold change estimates from each comparison (e.g. (3) vs (5)), and *s*_1_ and *s*_2_ are the corresponding standard deviation estimates (i.e. standard error multiplied by the square root of the number of samples) as reported by DESeq2. *n*_1_ and *n*_2_ are the number of samples total used for each analysis (e.g. for (3) vs (5), *n*_1_=2+3=5 and *n*_2_=90+102=192, see Table 1). When assessing significance, the degrees of freedom is calculated using the Welch-Satterthwaite equation:

$$\texttt{d.f.} = \frac{\left (\frac{s_{1}^{2}}{n_{1}} + \frac{s_{2}^{2}}{n_{2}} \right)^{2}} {\frac{(s_{1}^{2}/n_{1})^{2}}{n_{1}-1} + \frac{(s_{2}^{2}/n_{2})^{2}}{n_{2}-1}}. $$

### Comparison of DE gene lists and enriched gene sets

*t*-statistics were calculated as described above for (3) vs (5) and (1) vs (2), where positive *t* corresponded to an increased log2 fold change in HD+ CAU over HD+ BA9 and HD+ BA9 over HD BA9, respectively. For (3) vs (5), genes were then ranked by descending *t*-statistic and analyzed for gene set enrichment with fgsea [12]. These GSEA results were then combined with those calculated for (1), (2), and (4). Significantly enriched gene sets at FDR <0.05 were manually curated into 10 high level functional categories: Angiogenesis/Blood Brain Barrier (BBB), Apoptosis, Cell Cycle/Development, Cytoskeleton/Extracellular Matrix (ECM), Immune Response/Cancer, Metabolism, Neuron System, Protein Folding/Other, Signaling, and Transcription/Translation. Each gene set was also categorized into so-called Agreement Classes, an ordinal scale representing how specific the gene set is to HD+ CAU, as described in the “Results” section. Combination of GSEA results, curation of gene sets, calculation of agreement classes, and plots from Fig. 6 were made using python, jupyter lab, pandas, and matplotlib python libraries.

Gene sets within each functional category were also cast as a graph, where each node is a gene set, and edges between nodes were drawn if the gene sets shared more than 25% of their leading edge genes. Graph analysis was performed using python, networkx, and matplotlib. All analysis and figure code for this project are available at bitbucket.org/bubfnexus/asymptomatic_hd_mrnaseq .

## Results

### HD BA9, HD+ BA9, and HD+ CAU show concordant DE genes

**Fig. 2:**
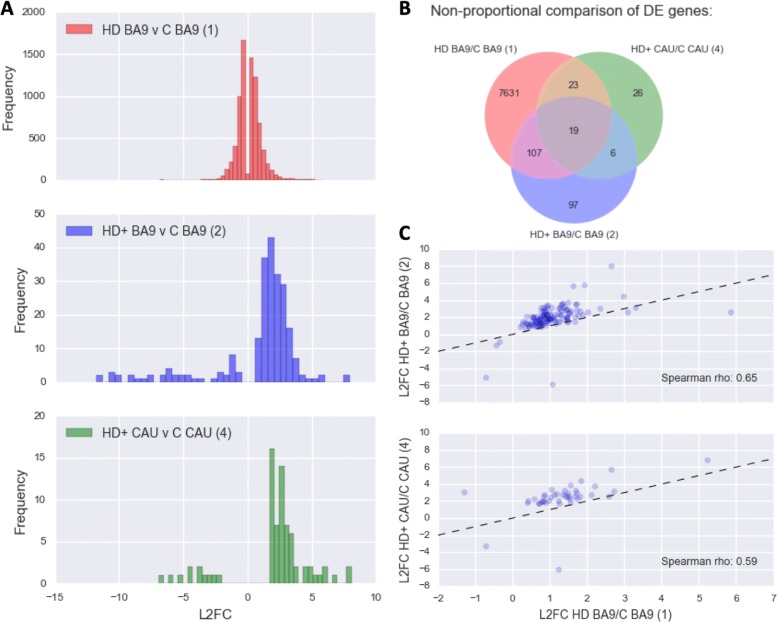
DE Genes from (1), (2), and (4). **a** Histograms of log2 fold changes **b** 3-way Venn diagram of significant DE genes **c** Scatter plots of log2 fold change in DE genes in (1) vs (2) and (1) vs (4) with Spearman *ρ*

Figure 2 contains differentially expressed (DE) gene metrics for analyses (1), (2), and (4). In Fig. 2a, we see that the fold change distribution is similar between all three analyses, where more genes have increased expression overall than decreased and that this is particularly evident in the HD+ versus control analyses. The overlap of DE genes at FDR <0.05 in Fig. 2b shows that analyses (1) and (4) are more similar to each other than to (2). Figure 2c depicts the similarity in log 2 fold change (L2FC) for the HD+ versus C in BA9 with HD versus C in BA9 (top figure) and the HD+ versus C in CAU with HD versus C in BA9 (bottom figure) for DE genes at *p*<0.05 in both groups. These two figures show the extent of similarity of L2FC across these different contrasts. It is interesting to note that the symptomatic HD BA9 expression profile is well correlated with the HD+ versus C in CAU (Spearman *ρ*=0.55) and consequently the HD BA9 appears to be a good model for early disease effects in HD. This concordance is particularly remarkable when considering that the numbers of samples in the HD+ vs C analyses are extremely small.

The overlapping DE genes in Fig. 2b provide insight into both common gene signatures across brain regions and disease state as well as those unique to individual conditions. Table 5 contains the DE statistics for the 19 genes found in the intersection of analyses (1), (2), and (4). These genes are perturbed across the entire disease course, from the HD+ BA9, which is the least affected tissue, to the most severely degenerated HD BA9 samples. All of these genes implicate the neuroinflammatory and neuroimmune responses, and seven of the 19 genes (BAG3, HSPA6, HSPB1, SERPINH1, DNAJB1, HSPA1A, HSPA1B) have direct roles in the heat shock response. As expected, the genes from Table 5 are highly enriched for unfolded protein binding, molecular chaperones and focal adhesion, heat shock response, apoptosis, and response to oxidative stress.

**Table 5:** Common response genes in HD BA9, HD+ BA9, and HD+ CAU, corresponds to middle intersection of Venn diagram in Fig. 2b

Symbol	Gene name	(1) BM	(1) L2FC	(2) BM	(2) L2FC	(4) BM	(4) L2FC
MAFF	MAFF BZIP TF	385.63	1.5	605.27	2.56	1672.23	2.55
NFIL3	Nuclear Factor IL3	510.65	1.02	737.64	1.83	1379.75	2.48
BAG3	BCL2 Associated Athanogene 3	1451.15	1.71	1808.36	2.87	10878.27	3.0
HSPA6	Heat Shock Protein Family A (Hsp70) Member 6	155.97	2.65	4821.76	7.99	19890.86	5.73
HSPB1	Heat Shock Protein Family B (Small) Member 1	5352.69	1.81	6311.86	2.68	26621.0	2.56
ANGPT2	Angiopoeitin 2	278.17	1.68	202.34	1.45	819.36	2.59
C5AR1	Complement C5a Receptor 1	104.03	1.46	201.91	2.91	647.85	2.47
SERPINH1	Serpin Family H Member 1	491.4	1.49	1432.76	3.77	6758.88	2.92
HILPDA	Hypoxia Inducible Lipid Droplet Associated	513.5	1.52	777.11	2.55	1946.98	2.27
GADD45B	Growth Arrest And DNA Damage Inducible Beta	1345.54	1.56	2470.43	2.76	6656.73	1.9
DNAJB1	DnaJ Heat Shock Protein Family (Hsp40) Member B1	3833.3	1.05	9591.0	3.16	33978.66	3.37
HSPA1A	Heat Shock Protein Family A (Hsp70) Member 1A	11803.6	1.69	24914.57	3.53	116944.49	3.27
PLIN2	Perilipin 2	360.3	0.84	477.77	2.21	1855.84	2.03
HSPA1B	Heat Shock Protein Family A (Hsp70) Member 1B	10324.61	1.39	20241.7	2.86	84872.14	3.31
GADD45G	Growth Arrest And DNA Damage Inducible Gamma	316.51	0.87	687.17	2.49	1374.17	1.88
ZC3H12A	Zinc Finger CCCH-Type Containing 12A	35.5	0.84	101.95	3.14	283.56	2.55
C10orf10	DEPP1, Autophagy Regulator	1764.55	1.12	1236.25	1.99	11253.83	2.75
RRAD	Ras Related Glycolysis Inhibitor And Calcium Channel Regulator	78.9	0.77	211.84	3.17	341.72	1.98
RGS16	Regulator Of G Protein Signaling 16	203.8	0.57	367.42	2.03	1198.58	2.24

Base mean columns are the mean normalized counts from the correspondng analysis. L2FC is log 2 fold change estimated by DESeq2. BM - base mean (number of normalized counts) for the gene

**Fig. 3:**
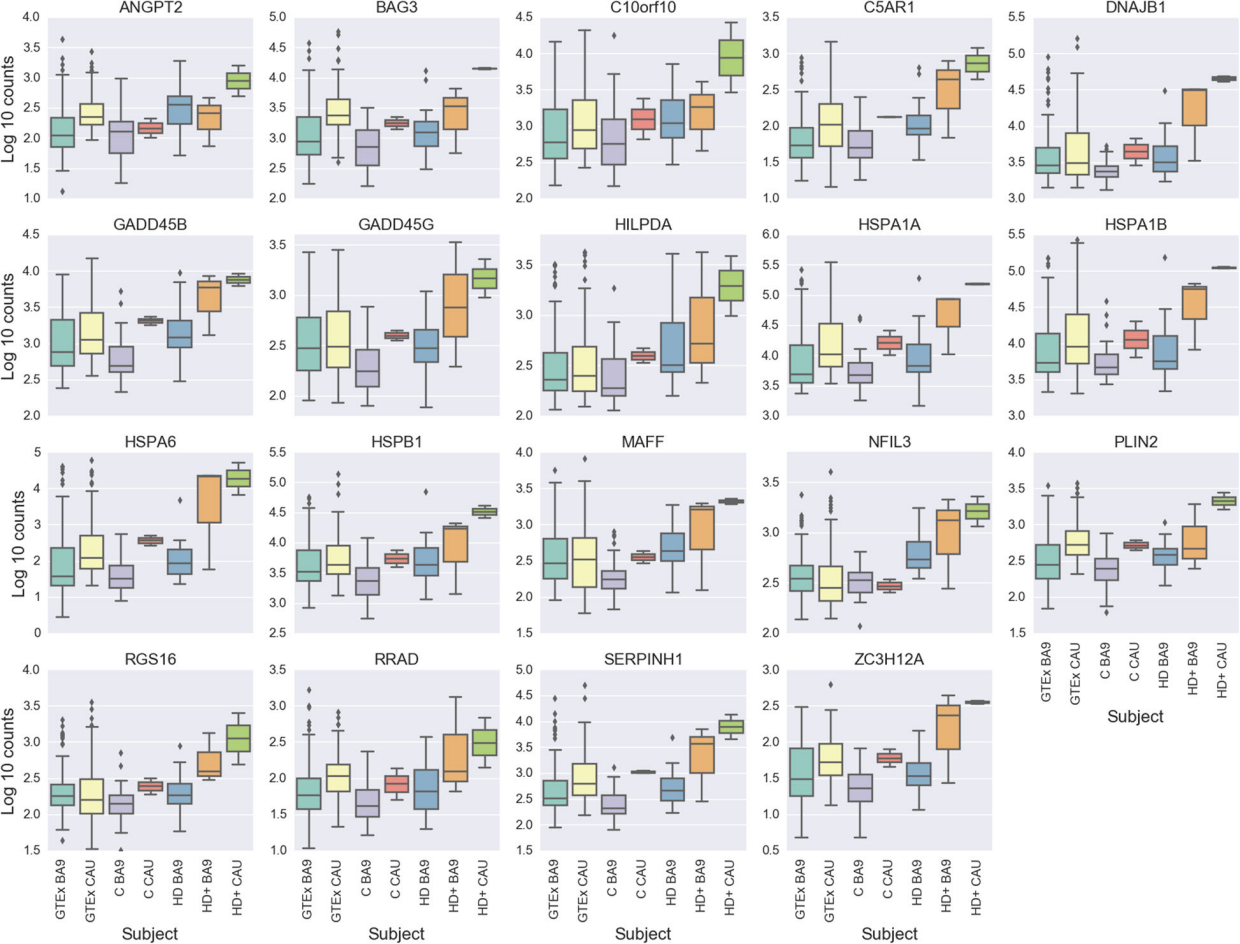
Boxplot of 19 common response genes in all analyses, corresponds to middle intersection of Venn diagram in Fig. 2b. Base mean columns are the mean normalized counts from all samples. The first two boxes correspond to GTEx BA9 and CAU, respectively, followed by the C BA9 and C CAU from this study. The last three in each plot depict HD BA9, HD+ BA9, and HD+ CAU, respectively

Figure 3 contains normalized counts distributions for each sample group for the 19 common DE genes from Fig. 2b. From left to right in each plot are counts from GTEx BA9, GTEx CAU, C BA9, C CAU, HD BA9, HD+ BA9, and HD+ CAU sample groups. Since there are so few C CAU samples in this study (i.e. only 2), we include the GTEx CAU counts (102 samples) to illustrate that our C CAU counts are well within the expected range for these genes. In every case except ANGPT2, the mean expression level increases from HD BA9 to HD+ BA9 to HD+ CAU. This increase in expression is particularly large for HSPA6, which shows a 256 fold abundance increase in HD+ CAU vs C CAU. Since the HD+ BA9 samples are the least affected tissues of the three disease groups, it is interesting to note that the asymptomatic HD+ BA9 samples show higher expression than the symptomatic HD BA9 samples overall for these genes.

The 26 genes that are uniquely DE in (4) from Fig. 2b (in green segment) appear in Table 6. These genes show only weak functional enrichment for extracellular space compartment, and plasma membrane, but we make two remarkable observations. First, several genes are consistent with the heat shock and inflammatory response observed in the common DE genes and in (1) more broadly, including HSPH1, CCL19, and CX3CR1. Second, four of the genes are readthrough transcripts (RPS10-NUDT3, UBE2F-SCLY, RPL17-C18orf32, and RP5-850E9.3) that originate from different chromosomes.

**Table 6:** Unique response genes in HD+ CAU, corresponds to only the green area diagram in Fig. 2b

Symbol	Gene name	(1) BM	(1) L2FC	(2) BM	(2) L2FC	(4) BM	(4) L2FC
CDHR4	Cadherin Related Family Member 4	3.93	-0.92	16.52	-1.0	47.49	-4.6
SIK1	Salt Inducible Kinase 1	172.19	0.96	351.77	2.86	452.63	3.11
CH507-9B2.1	Uncharacterized ncRNA gene	202.22	-0.98	172.02	2.0	292.54	5.39
TMIE	Transmembrane Inner Ear	286.83	-0.21	387.23	0.07	380.24	2.33
DHFRP1	Dihydrofolate Reductase Pseudogene1	ND	ND	ND	ND	21.19	7.87
RPS10-NUDT3	Read-through transcript	247.15	0.29	297.06	0.59	386.09	2.01
RSC1A1	Regulator Of Solute Carriers 1	ND	ND	24.01	4.78	41.98	5.89
CCL19	C-C Motif Chemokine Ligand 19	34.45	-0.21	13.66	-2.58	166.21	-4.54
LRRC71	Leucine Rich Repeat Containing 71	11.97	0.61	33.61	0.13	67.87	-3.71
HSPH1	Heat Shock Protein Family H Member 1	9773.91	0.25	13966.0	0.6	20222.49	1.72
CX3CR1	C-X3-C Motif Chemokine Receptor 1	338.16	0.08	389.08	-0.81	472.45	-2.87
UBE2F-SCLY	Read-through transcript	31.89	-0.18	45.88	0.83	48.19	4.65
NSFP1	N-Ethylmaleimide-Sensitive Factor Pseudogene 1	15.47	-1.69	51.57	-0.15	23.53	8.09
RPL17-C18orf32	Read-through transcript	275.3	-0.13	417.4	-0.36	523.12	2.23
THBS1	Thrombospondin 1	205.54	0.45	347.57	2.02	1448.89	2.64
DYDC2	DPY30 Domain Containing 2	51.37	-0.74	154.16	-1.57	251.82	-2.49
CBSL	Cystathione-Beta-Synthase Like	754.57	0.3	746.02	2.38	789.33	3.06
PTGS2	Prostaglandin-Endoperoxide Synthase 2	313.94	0.01	390.51	1.64	291.52	2.1
LINC00473	Long Intergenic Non-Protein Coding RNA 473	60.5	0.41	68.35	1.52	75.01	3.55
CCDC33	Coiled-Coil Domain Containing 33	9.19	-1.08	37.91	-1.46	60.08	-5.15
SLC38A5	Solute Carrier Family 38 Member 5	505.13	-0.12	530.55	-0.83	641.39	-2.4
NPIPB15	Nuclear Pore Complex Interacting Protein Family Member B15	177.05	0.61	112.27	-3.21	231.41	-3.9
NPIPA8	Nuclear Pore Complex Interacting Protein Family Member A8	345.69	0.52	256.3	-1.61	82.36	-6.91
LTF	Lactotransferrin	45.19	-0.52	152.43	-0.91	1448.49	3.24
RPL10P9	Ribosomal Protein L10 Pseudogene 9	29.12	0.64	76.79	2.08	107.51	6.1
RP5-850E9.3	Read-through transcript	106.96	-0.13	175.87	0.45	78.98	4.96

Base mean columns are the mean normalized counts from the corresponding analysis. L2FC is log 2 fold change estimated by DESeq2. FDR <0.05 are considered significant for analyses (1) and (2). BM - base mean (number of normalized counts) for the gene. ND - genes not detected or too lowly abundant for consideration in the corresponding samples

### HD+ vs C CAU enriched pathways are a subset of those in HD BA9

**Fig. 4:**
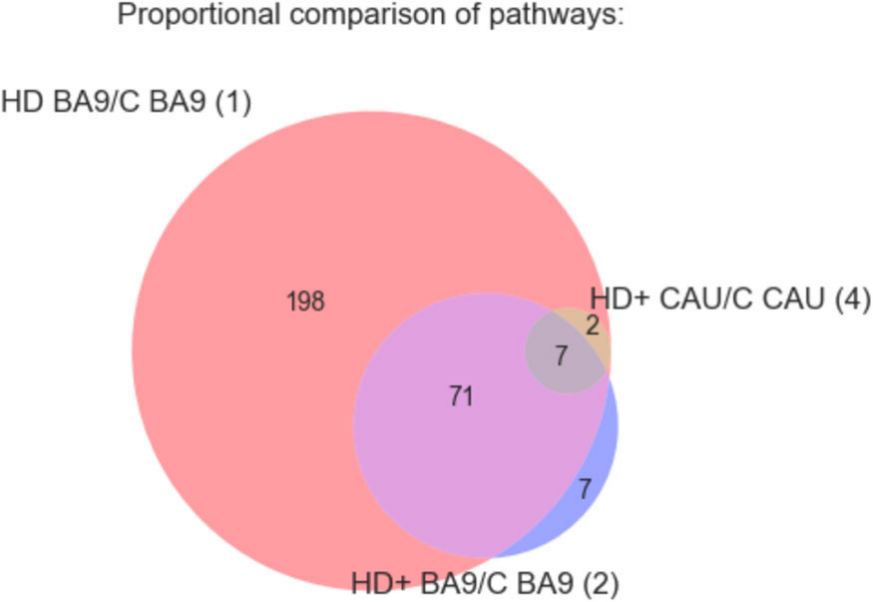
GSEA results for DE genes from analyses (1), (2), and (4). Figure shows overlap of significantly enriched MSigDB C2 Canonical Pathway gene sets at *p*_*adj*_<0.05 irrespective of direction of effect. Selected gene sets are included in Table 7 and the full results are in Additional file 4

We next performed gene set enrichment analysis on each DE gene list to identify associated biological functions. Figure 4 contains the result of gene set enrichment analysis from analyses (1), (2), and (4) using the GSEA [14] algorithm as implemented in the fgsea R package [12] against the MSigDB C2 Canonical Pathway gene set database[14, 15]. Analysis (1), which has the most power to detect DE genes identifies 195 significantly enriched pathways at FDR <0.05. All 13 of the pathways identified in the HD+ CAU versus C CAU are among these 195 of (1), and eleven of these are also seen in the HD+ BA9 versus C BA9. The substantial overlap of the enriched pathways suggests that the most highly perturbed pathways in the prodromal phase of disease expression are also detected in late stage HD BA9. Only seven pathways, seen in the HD+ BA9 versus C BA9 are not also seen in (1). Table 7 lists the 16 gene sets that are significantly enriched in either both (1) and (4) (9 gene sets) or are unique to (2) (7 gene sets). Consistent with our previous work [4], the enriched pathways heavily implicate an increase in neuroimmune and neuroinflammatory response, an increase in transcriptional activity, and a decrease in neuron-related pathways.

**Table 7:** Significantly enriched pathways in intersection of (1) and (4) or unique to (2) from Fig. 4

Pathway	(1) NES	(2) NES	(4) NES
KEGG SYSTEMIC LUPUS ERYTHEMATOSUS	1.76	2.56	2.29
PID SMAD2 3NUCLEAR PATHWAY	1.91	2.24	1.85
PID P53 DOWNSTREAM PATHWAY	1.86	2.26	1.84
PID AP1 PATHWAY	1.8	2.1	2.03
REACTOME HEMOSTASIS	1.46	1.87	1.46
KEGG CYTOKINE CYTOKINE RECEPTOR INTERACTION	1.52	2.56	1.76
REACTOME INNATE IMMUNE SYSTEM	1.52	2.16	1.4
BIOCARTA CK1 PATHWAY	NS	-1.88	NS
REACTOME INSULIN SYNTHESIS AND PROCESSING	NS	-1.85	NS
PID REG GR PATHWAY	NS	1.56	NS
KEGG GLYCOSAMINOGLYCAN BIOSYNTHESIS HEPARAN SULFATE	NS	-1.75	NS
REACTOME NEUROTRANSMITTER RELEASE CYCLE	NS	-1.7	NS
KEGG ARACHIDONIC ACID METABOLISM	NS	1.58	NS
PID HNF3A PATHWAY	NS	1.61	NS
REACTOME POTASSIUM CHANNELS	-1.78	NS	-1.74
BIOCARTA NFAT PATHWAY	1.95	NS	1.87

NES = normalized enrichment score from GSEA, where positive or negative values indicate the genes in the pathway are increased or decreased, respectively, in disease compared with control. NS = not significant

### HD BA9 DE genes perfectly predict disease state in HD+ CAU

**Fig. 5:**
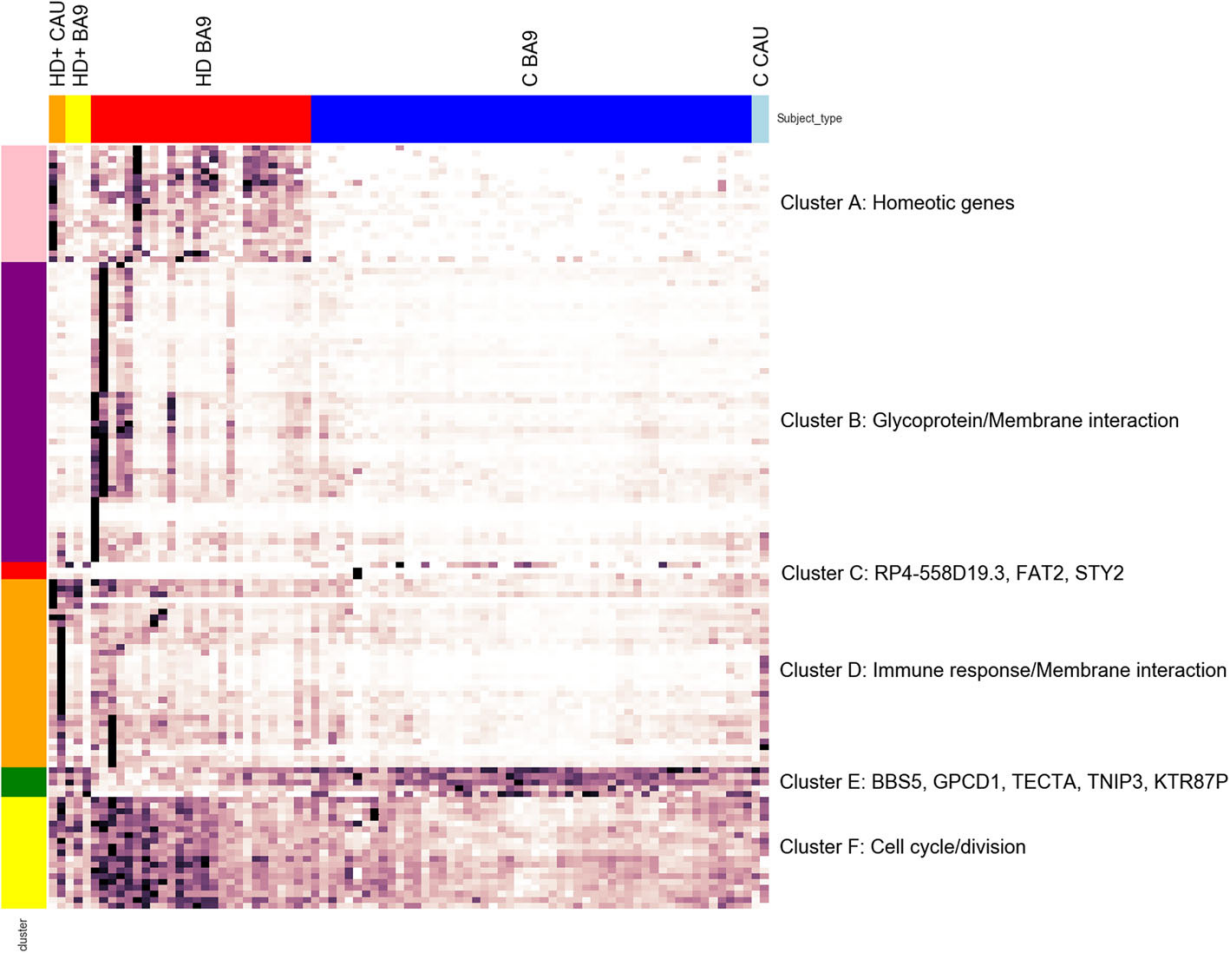
Clustered heatmap of normalized counts of top 200 genes from (1). Row clusters were created manually by inspection guided by clustered dendrogram, and enriched biological pathways (or the genes themselves) for the genes in each cluster are listed as indicated. Color label: HD+ CAU - orange, HD+ BA9 - yellow, HD BA9 - red, C BA9 - blue, C CAU - light blue

Figure 5 shows the normalized counts from all HD, HD+, and C samples for the top 200 genes found to be DE in analysis (1) as a heatmap. A distinctive result from our previous HD work [4] was that a set of homeotic genes, most notably the HOX gene clusters, were selectively increased in HD compared with C. By inspection, HD+ CAU appears to demonstrate similar homeotic gene expression to HD, suggesting that the disease process in asymptomatic caudate does indeed resemble symptomatic cortex in these samples. HD+ BA9 expression in these genes is less pronounced and more closely resembles C samples, further supporting the hypothesis that the effect of disease on BA9 is reduced in HD+ individuals. The results suggest that HD+ CAU is more similar to HD BA9, and HD+ BA9 is more similar to C.

We sought to perform a more unbiased analysis to better quantify the similarity of the HD+ samples to either HD or C by training a random forest decision tree classifier on the HD and C samples. Briefly, a decision tree classifier identifies key features (in our case these are genes) that partition labeled samples (here either HD or C) into like groups using a threshold cutoff for each gene. A decision tree built using a dataset can then be used to predict the class of new samples that were not used to build the tree. To avoid overfitting, the random forest algorithm generates many different decision trees by randomly sampling samples and genes with replacement many times. When applied to a new sample, the output of a random forest decision tree classifier is the number of trees that predicted the sample to have each label. A random forest where all trees classify a new sample to have the same label indicates a perfect classification. A random forest predicting a sample to be of either class with equal frequency has no predictive power. See the “Methods” section for more details on the random forest decision tree algorithm.

After creating the random forest based on the top 250 significant genes in (1), the forest was used to predict the sample type of each HD+ BA9 and CAU sample. The results of the classifier are in Table 8. Several aspects from the random forest results are of note. First, the random forest perfectly classified both the HD+ CAU samples as symptomatic HD BA9, supporting the intuition built from the heatmap in Fig. 5 (Table 8(a)). Second, the HD+ BA9 samples were evenly split between being predicted as HD BA9 and C BA9 (Table 8(a)). This suggests that there are some genes in the HD+ BA9 samples that resemble symptomatic HD BA9, and others that more closely resemble control BA9. We will explore this difference in greater detail in the last section. Third, there is high prediction consistency for HD+ CAU even when choosing 250 genes randomly from (1), and a greater agreement in classifying HD+ BA9 as C BA9 (Table 8(b)). These results suggest that the DE signal for HD+ CAU and in HD BA9 is strong and genome wide, and are consistent with the hypothesis that HD+ BA9 represents a less severe form of the same response as in HD+ CAU and HD BA9. Last, when the model is fully randomized (i.e. random genes and shuffled labels from (1), Table 8(c)), classification consistency is essentially random, consistent with our expectation of the model. Taken together, this unbiased classification analysis supports the hypothesis that changes in BA9 after symptoms have appeared are reflected in the asymptomatic HD+ caudate and, to a lesser degree, in HD+ BA9.

**Table 8:** Random forest decision tree classifications of HD+ using genes from (1)

(a) Trees built with top 250 DE genes from (1)		
Top	HD BA9	Control BA9
HD+ BA9	0.354	0.646
HD+ CAU	1	0
(b) Trees built with 250 random genes from (1)		
Random	HD BA9	Control BA9
HD+ BA9	0.318	0.682
HD+ CAU	0.940	0.060
(c) Trees built with 250 random genes from (1) and shuffled labels		
Null	HD BA9	Control BA9
HD+ BA9	0.485	0.515
HD+ CAU	0.464	0.536

All figures are the fraction of 20,000 trees that predicted each sample to have the corresponding label indicated in the column. E.g. 49.5% of the trees predicted HD+ BA9 samples to be HD BA9

### Gene expression patterns and pathways unique to HD+ CAU

Understanding the factors that cause the caudate to degenerate first in HD is critical in understanding the HD disease process. Due to the small number of HD+ and C CAU samples (2 and 3, respectively), the DE statistics for this direct comparison is likely to be highly influenced by noise, as evidenced by the small number of enriched gene sets in this comparison seen in Fig. 4. In addition, directly comparing HD+ CAU and HD+ BA9 may reveal differences between brain regions that are not prominent when comparing the results of corresponding pairwise comparisons. We therefore devised a statistical strategy to identify genes and pathways that are as robust and specific to HD+ CAU as possible. We therefore devised a statistical strategy to identify genes and pathways that are as robust and specific to HD+ CAU as possible.

Since disease status and brain region are convolved in the DE genes identified in (3), we sought to identify genes that differ between CAU and BA9 due to the disease process, and not due to differences in brain region. To accomplish this, the DE results from (3) and (5) were compared by computing a t-statistic of the difference in log2 fold change estimates and their standard errors reported by DESeq2 (see “Methods” section). In essence, this statistical procedure quantifies the difference in log2 fold change of genes when comparing HD+ CAU versus HD+ BA9 while de-emphasizing genes that are different due to differences in brain region. The resulting statistics allow genes to be ranked by the degree of relevance to the disease process in HD+ CAU. Table 9 contains the top 10 genes ranked by descending absolute value of the t-statistic to illustrate this strategy. For example, CFAP157 is increased 19.6 (2^4.3^) fold in GTEx CAU over GTEx BA9, but is decreased by 1.09 (2^−0.13^) fold in HD+ CAU over HD+ BA9, resulting in an difference in fold change of -4.43 (i.e. −0.13−4.3=−4.43). TVP23C-CDRT4, another readthrough transcript, is essentially unchanged in GTEx CAU compared with GTEx BA9, but is increased 5.85 fold in HD+ CAU over HD+ BA9 (2.55−(−0.02)=2.57).

**Table 9:** Top 10 genes that show different effect sizes (L2FC) between (3) and (5)

ENSGID	Gene symbol	HD+ L2FC (3)	GTEx L2FC (5)	*Δ* (3) vs (5) L2FC	*t*
ENSG00000160401	CFAP157	-0.13	4.30	-4.43	20.45
ENSG00000077327	SPAG6	-2.20	2.64	-4.84	15.28
ENSG00000152611	CAPSL	-2.59	3.65	-6.24	14.63
ENSG00000181085	MAPK15	-1.65	2.71	-4.36	12.89
ENSG00000154914	USP43	-3.32	-0.80	-2.52	12.87
ENSG00000169436	COL22A1	-3.82	-0.31	-3.51	12.82
ENSG00000259024	TVP23C-CDRT4	2.55	-0.02	2.57	-12.75
ENSG00000162747	FCGR3B	3.21	0.31	2.90	-12.45
ENSG00000118113	MMP8	4.70	0.13	4.57	-12.43
ENSG00000103569	AQP9	1.59	-0.72	2.31	-12.41
ENSG00000140795	MYLK3	-0.78	1.87	-2.65	12.10

These genes are most likely perturbed in CAU specifically due to HD and not due to brain region. *Δ* L2FC is the log2 fold change of (5) minus (3), where a positive value means that gene expression is greater in HD+ CAU vs BA9 than GTEx CAU vs BA9. Full results are in Additional file 5

**Fig. 6:**
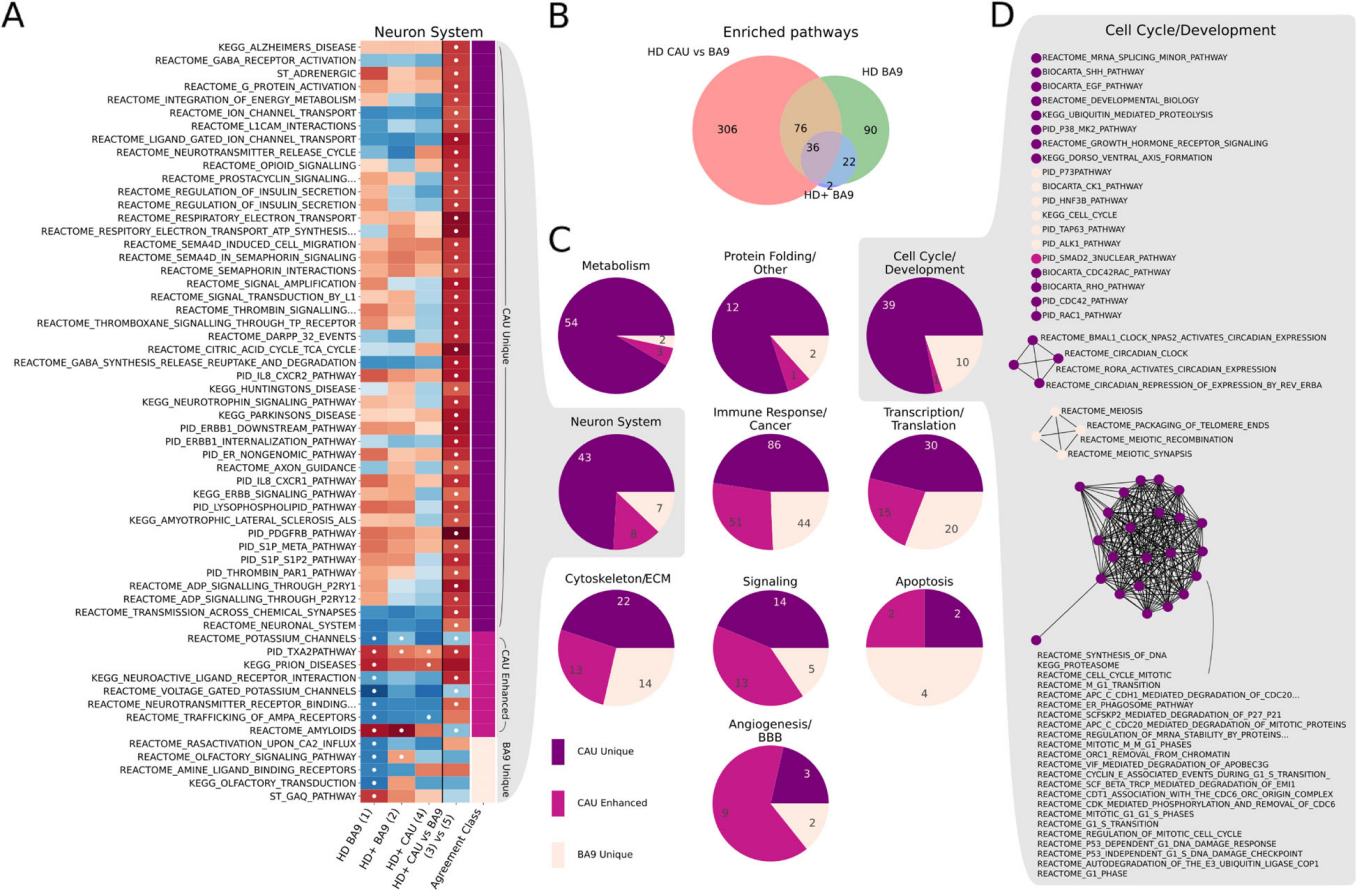
**a** Enriched gene sets related to the Neuron System. The first four columns plot Normalized Enrichment Score (NES) of GSEA analyses from (1), (2), (4), and (3) vs (5), respectively, where red and blue correspond to positive and negative NES scores, respectively. The fifth column indicates the Agreement Class of each gene set, assigned according to HD+ CAU-specificity. Cells with white dots indicate that gene set is significantly enriched in the corresponding analysis. **b** Overlap of significantly enriched gene sets regardless of category. The gene sets enriched in (4) are a subset of those in (1), and thus are not listed. **c** Distribution of gene sets by agreement class divided into ten high level functional categories, showing that some functions are more selectively enriched in HD+ CAU relative to BA9 than others. **d** Graph-based representation of the Cell Cycle/Development gene sets from C. Each node is a gene set, and nodes with connected edges share more than 25% of their leading edge genes, thus representing the same expression signal

The resulting t-statistics from this analysis induced a ranking of genes that were then subjected to gene set enrichment analysis against the MsigDB C2 Canonical Pathway gene set database. The analysis identified 405 significantly enriched gene sets at FDR <0.05, and all but one of these gene sets were positively enriched, indicating that genes increased in HD+ CAU relative to HD+ BA9 have strong functional coherence (full fgsea results in Additional file 3). These results were combined with the enriched gene sets from (1), (2), and (4) and subsequently divided into so-called Agreement Classes based on the pattern of significance across all four analyses. The Agreement Class is an ordinal indicator for the degree of HD+ CAU-specificity as follows. CAU Unique gene sets are only seen in HD+ CAU relative to HD+ BA9 (i.e. (3) vs (5)). CAU Enhanced are enriched gene sets in HD BA9 vs C BA9 (1) as well as in either HD+ CAU vs C CAU (4) or HD+ CAU relative to HD+ BA9 ((3) vs (5)). Finally, BA9 Unique only show enrichment in HD BA9 vs C BA9 (1). To aid in interpretation, the gene sets were manually curated into 10 high level functional categories: Angiogenesis/Blood Brain Barrier (BBB), Apoptosis, Cell Cycle/Development, Cytoskeleton/Extracellular Matrix (ECM), Immune Response/Cancer, Metabolism, Neuron System, Protein Folding/Other, Signaling, and Transcription/Translation. To illustrate these ideas, a heatmap of the enriched gene sets related to the Neuron System is in Fig. 6a.

As seen in Fig. 6b, 306 out of 405 significantly enriched gene sets are unique to CAU relative to BA9. The distribution of these unique gene sets varies by biological process (Fig. 6c), where processes related to Cell Cycle/Development, Metabolism, Neuron System, and Protein Folding/Other show the greatest proportion of CAU-unique gene sets. This is in contrast to Angiogenesis/BBB, where most of the gene sets are seen in both CAU relative to BA9, and in BA9 independently. Gene sets related to Apoptosis, Cytoskeleton/ECM, Immune Response/Cancer, Signaling, and Transcription/Translation have a mixture of CAU unique, CAU enhanced, and BA9 specific gene sets. When we examine the enriched gene sets from Cell Cycle/Development more closely using a graph-based representation (Fig. 6d), we observe that there are two distinct groups of genes enriched separately in CAU vs BA9 and BA9 itself. In particular, BA9 is enriched for a set of genes relating to meiosis, whereas the CAU unique processes involve mitosis. Heatmaps and graph representations of all other categories are included in Additional file 1. Overall, this comparison of enriched gene sets in HD+ CAU relative to BA9 with the other two brain regions identifies the common and different cellular processes that are active in different brain regions.

### Comparing DE gene lists identifies early vs late responding genes

In the random forest analysis discussed above, we noted that the HD+ BA9 samples were classified either as HD BA9 or C BA9 with approximately equal frequency. This suggests that there are some genes with an expression pattern that resembles HD BA9 and some that are yet unaffected in asymptomatic HD+ BA9. Thus, the genes that are consistent between HD+ BA9 and HD BA9 are genes that may form an early response in HD, whereas the genes whose expression differs from HD BA9 might still be intact and only respond later in the disease. We sought to identify which genes were early vs late responders by applying our t-statistic strategy comparing log2 fold changes between analyses (1) and (2). Table 10 contains results from the t-statistic based analysis of (1) and (2).

**Table 10:** Genes partitioned by significance within analyses (1) and (2) and fold change difference between these analyses

	(1) HD vs C BA9		(2) HD+ vs C BA9		
	DE	Not DE	DE	Not DE	Total
Sig. Between	4454	11670	218	15906	32248
Not Sig. Between	3281 (54)	12634 (387)	9 (2)	15906 (3765)	31830
Total	7735	24304	227	31812	
Grand total	32039		32039		

Numbers in parentheses are genes that appeared in the corresponding analysis but were filtered out in the other

**Fig. 7:**
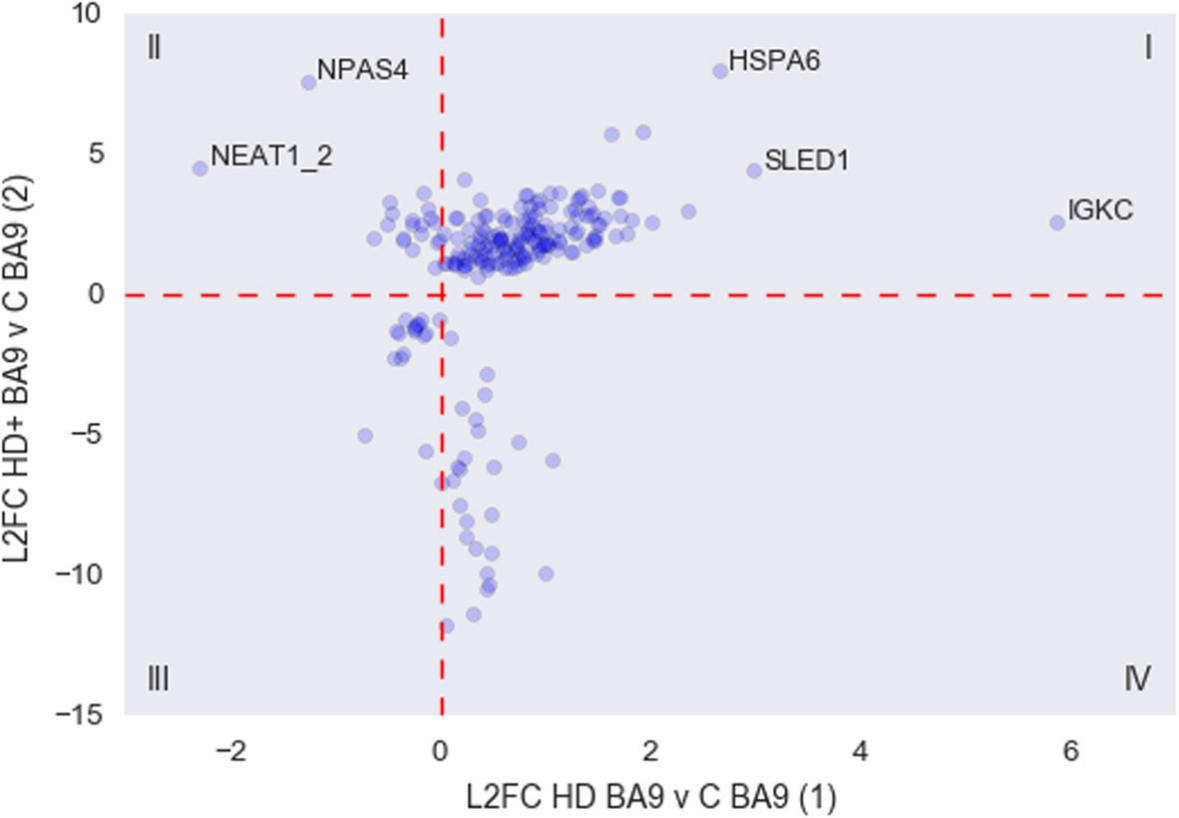
Scatter plot of fold changes from 215 early response genes from (1) and (2)

Of particular note are the 215 genes that are DE in (2) and have fold changes different from (1). These are the genes that may reflect early disease processes not identifiable in symptomatic individuals post mortem. We extracted these 215 genes and plotted their log fold changes to examine the relationship between groups as depicted in Fig. 7. Genes in quadrants I and III of the figure are genes that show the same direction of effect (i.e. up or down) but have a different size of effect. Genes in quadrants II and IV are genes that show differential behavior, and are thus potentially unique responses early in the disease process that are not observed in symptomatic HD BA9.

The statistics from the genes in quadrants II and IV, as well as the 4 additional genes detected in (2) that were filtered out of (1) due to low counts are listed in Tables 11 and 12. Most of the genes that are down regulated in (2) with respect to (1) are ribosomal protein genes that are essentially absent from HD+ BA9. The two genes that are massively increased in (2) but decreased in (1) are NPAS4, Neuronal PAS Domain Protein 4, and NEAT1_2.

**Table 11:** Putative early response genes in HD+ BA9 from Fig. 5 quadrant II

Ensembl ID	Gene name	(1) Basemean	(1) L2FC	(2) Basemean	(2) L2FC
ENSG00000125740	FOSB	363.46	-0.17	1438.94	3.63
ENSG00000174576	NPAS4	165.91	-1.27	2473.17	7.64
ENSG00000278050	NEAT1_2	2.51	-2.29	14.4	4.57
ENSG00000135625	EGR4	263.89	-0.48	832.95	3.35
ENSG00000173391	OLR1	229.69	-0.05	566.64	2.62
ENSG00000198576	ARC	897.43	-0.51	2278.26	2.54
ENSG00000158050	DUSP2	308.83	-0.28	850.53	2.54
ENSG00000153234	NR4A2	519.76	-0.18	1049.01	2.21
ENSG00000120738	EGR1	1826.3	-0.64	3887.13	2.06
ENSG00000248713	RP11-766F14.2	31.18	-0.37	71.78	1.96
ENSG00000160223	ICOSLG	796.19	-0.36	813.93	2.03
ENSG00000232352	SEMA3B-AS1	30.91	-0.27	33.01	1.67
ENSG00000174429	ABRA	14.35	-0.11	35.74	2.78
ENSG00000273186	RP11-339B21.10	9.21	-0.46	10.63	2.95
ENSG00000122877	EGR2	136.81	-0.27	366.35	2.72
ENSG00000123358	NR4A1	1771.51	-0.19	4397.13	2.52
ENSG00000162783	IER5	1079.98	-0.02	2118.33	2.0
ENSG00000105722	ERF	968.7	-0.05	1248.28	1.01
ENSG00000244062	RP11-404G16.2	31.01	-0.13	52.64	3.11
ENSG00000184378	ACTRT3	37.62	-0.05	81.25	1.9

Base mean columns are the mean normalized counts from the corresponding analysis. L2FC is log 2 fold change estimated by DESeq2

**Table 12:** Putative early response genes in HD+ BA9 from Fig. 5 quadrant IV

Ensembl ID	Gene name	(1) Basemean	(1) L2FC	(2) Basemean	(2) L2FC
ENSG00000258017	RP11-386G11.10	7775.65	0.25	3679.44	-8.64
ENSG00000176868	RP11-334J6.7	2651.11	0.43	1455.75	-10.47
ENSG00000255082	GRM5-AS1	2209.09	0.19	1407.72	-6.19
ENSG00000254873	RP11-770J1.5	980.14	0.18	620.42	-7.46
ENSG00000267469	AC005944.2	2028.65	0.43	1058.48	-9.89
ENSG00000269604	AC005523.2	1008.72	0.06	565.69	-11.78
ENSG00000272379	RP1-257A7.5	697.88	0.47	385.27	-7.77
ENSG00000225339	RP11-513I15.6	5979.33	0.11	3352.43	-6.56
ENSG00000265401	RP11-138I1.4	4353.79	0.23	2305.67	-5.75
ENSG00000232940	HCG25	272.52	0.42	137.87	-3.5
ENSG00000271127	LL22NC03-N64E9.1	26.44	0.34	22.19	-4.43
ENSG00000273489	RP11-180C16.1	1300.83	0.5	737.27	-6.13
ENSG00000228748	RP13-39P12.3	301.31	0.44	190.55	-2.79
ENSG00000233427	RP1-212P9.3	28.1	0.75	13.53	-5.25
ENSG00000269145	AC007192.6	761.47	0.3	390.97	-11.38
ENSG00000279753	AC011558.5	15.79	1.07	6.54	-5.88
ENSG00000261641	LA16c-390E6.5	296.63	0.99	132.13	-9.85
ENSG00000268220	RP11-379K17.12	505.97	0.33	317.65	-8.97
ENSG00000269243	CTD-2231E14.8	214.65	0.15	109.74	-6.07
ENSG00000267436	AC005786.7	95.9	0.24	43.6	-8.07
ENSG00000279767	AL513523.2	642.42	0.46	288.95	-10.33
ENSG00000258430	RP11-982M15.2	210.57	0.47	103.36	-9.17
ENSG00000219410	RP4-761J14.8	363.25	0.36	195.65	-4.79
ENSG00000120992	LYPLA1	625.77	0.1	563.93	-1.49
ENSG00000256341	RP11-21A7A.3	23.56	0.21	15.33	-4.01
ENSG00000249141	RP11-514O12.4	Na	Na	4.87	5.35
ENSG00000279909	AC110615.1	Na	Na	187.8	-10.4

Base mean columns are the mean normalized counts from the corresponding analysis. L2FC is log 2 fold change estimated by DESeq2

## Discussion

To the authors knowledge, this is the first genome-wide transcriptome analysis of post-mortem asymptomatic HD+ BA9 and CAU. It is also the first systematic comparison of post-mortem symptomatic (HD) BA9 with asymptomatic (HD+) BA9 and CAU gene expression. Differential expression (DE) analysis identified many genes that show altered abundance between diseased and control tissue across brain regions, and there is a high degree of concordance in the direction of effect for these genes. The genes that are commonly DE in HD BA9, HD+ BA9, and HD+ CAU are strongly enriched for heat shock response, while the DE genes specific to HD+ CAU contain some heat shock elements and read-through transcripts. Gene set enrichment results show a high degree of agreement between these three analyses.

The analysis comparing HD+ CAU to HD+ BA9, and filtered using GTEx data for genes specific to brain region, identified a strikingly large number of significantly enriched gene sets that suggest processes related to Cell Cycle/Development, Metabolism, Neuron System, and Protein Folding are the most uniquely perturbed in the HD+ CAU disease process. Overall this analysis suggests that while a large proportion of disease processes are shared between CAU and BA9, there are distinct and important sets of genes perturbed in each brain region related to the disease process. Nonetheless, when the HD+ samples are classified using a random forest classifier built using the symptomatic BA9 samples, there is complete consensus that HD+ CAU most closely resembles HD BA9, while HD+ BA9 resembles aspects of both diseased and control brain. The homeotic and inflammatory gene signatures appear to be equally present in the HD+ CAU and HD BA9, suggesting a similar process affects the cellular milieu in both brain regions.

Finally, we identified key genes that appear to be early responders to the disease process by comparing HD+ BA9 and HD BA9. HD+ BA9 appears to be the least affected brain region of the three studied here; therefore, genes that show different behavior in these regions are likely to be part of an early response that is lost as the disease process progresses. The two genes in particular that are increased in HD+ BA9 relative to HD BA9 are NPAS4, which as been implicated in the cortex of mouse models of HD [16] and NEAT1, which has been shown to be associated with neuronal hyperactive state [17]. A second group of poorly annotated but consistently expressed genes seems to be uniquely expressed in HD BA9 and may be evidence of severe transcriptional dysregulation previously observed in this tissue[3, 4].

The authors caution that, due to small sample size, the results from HD+ BA9 and CAU should be best interpreted as trends warranting further study. Nonetheless, despite the small HD+ sample size, the consistency between the HD+ and HD BA9 results supports the robustness of these findings. Not only do the overall effect size and enriched pathway signatures agree to a great extent, many of the biological processes implicated are well supported in the literature. Immune response has been heavily implicated in HD and neurodegenerative disease in general[3, 4, 18–22], and the broad agreement between the diseased tissues across brain regions in this study lends support to the role of inflammation in the prodromal HD brain. Of particular note is the common heat shock response observed in the common DE genes in all comparisons with control. The heat shock system is primarily responsible for maintaining proteostasis and protein conformation during times of stress, and has been directly implicated in both animal [23] and in vitro [24] models of Huntington’s disease. The fact that expression of key heat shock genes appears to be perturbed across the entire disease course is strong evidence of the important role these proteins play in disease.

The genes found to be perturbed across the entire disease course provide an opportunity to develop prognostic tests for HD progression. It is interesting to note that the mRNA abundance of the commonly perturbed genes is often increased in HD+ BA9 relative to HD BA9, suggesting that protein levels of these genes might be usefully measured in a longitudinal setting to track disease progression. HSPA6 in particular shows a massive induction in HD+, and is an attractive first target for follow-up in a clinical setting. However, this gene is but one of thousands implicated by this analysis, and the authors urge readers to avoid the temptation to focus too specifically on any one gene reported here. A robust clinical test measuring disease progression will likely take the form of a panel of key inflammatory and possibly developmental genes measured longitudinally from a peripheral source such as blood or cerebrospinal fluid.

The differences revealed between HD+ CAU and HD+ BA9 may offer insight into why the striatum is uniquely vulnerable to neurodegeneration. The enriched functional categories that are the most specific to HD+ CAU include Metabolism and Cell Cycle and Development. Interestingly, when the cell cycle gene sets are examined closely (Fig. 6d), we observe that the gene sets uniquely enriched in HD+ CAU are related to mitosis, while the smaller number enriched in BA9 involve meiosis. The striatum, unlike the cortex, has a resident population of neuroblasts that enables neurogenesis in the adult human brain[25]. A recent hypothesis has proposed that these neuroblasts are impaired in HD, resulting in a lack of repleneshing neurons over time and eventual destruction of tissue [26]. The unique presence of increased mitotic gene expression, paired with the observation that many neuronal pathways are also increased in HD+ CAU compared with HD+ BA9, is strong evidence that neurogenesis is indeed active in this region prior to symptom onset. However, it still remains to be shown why these specific neurons degenerate in the first place, and why this neurogenesis ceases over time. The enrichment of meiosis in BA9 is curious, and does not lend an immediate interpretation. One possibile explanation is that the same signals that trigger neurogenesis in CAU are also present in BA9, but that cortical neurons lack neurogenic capabilities and ’misfire’ in response to the developmental signals. An intriguing feature of the HD BA9 samples is the expression of homeotic and developmental genes, which might be a consequence of a neuron that is trying to regenerate but cannot.

Given the extreme rarity of HD+ CAU samples, it is difficult to conceive of validation experiments to test these findings given our current disease models. The combined complexity of the central nervous and immune systems makes accurate models of human HD challenging to devise, since there is clear involvement and interaction of major players in both of these systems. Given the immense complexity of both the human HD+ and HD phenotype, the authors also urge caution in drawing strong mechanistic lines between any small number of genes reported here and the disease process. With the exception of the few putative early responder genes identified, it is essentially impossible to separate the causes and effects of neurodegeneration from this inherently observational study. Only a few of the many findings in this study have been discussed in this manuscript, and much greater insight may likely be gained from further examination by those specializing in different aspects of the biology implicated here. These results are therefore put forward as a source of hypothesis and inspiration for new models and avenues of research.

## Conclusion

These results provide clear evidence that the caudate nucleus is strongly affected by the HD disease process prior to the appearance of any symptoms. The common gene expression patterns observed in asymptomatic HD+ CAU compared with symptomatic HD BA9 supports the hypothesis that many aspects of the disease process are shared between brain regions, but that the CAU experiences these changes at a much earlier stage of disease than BA9. Of particular importance is the set of heat shock related genes that is commonly perturbed across all HD samples. Despite these similarities, a much broader set of gene expression changes is observed specifically in HD+ CAU, implicating unique disease processes that may help explain why this brain region is particularly sensitive to degeneration. Several pieces of evidence suggest that neurogenic signals are present in both CAU and BA9, but that the response to these signals differentiates between region. The nature and significance of this putative neurogenic signal remains to be shown, and should be investigated further along with the other CAU-specific gene expression patterns found by this study.

## Supplementary information

**Additional file 1** Supplemental File 1. Heatmaps and clustered gene set networks for biological categories from Fig. 6.

**Additional file 2** PCA Outlier Analysis

**Additional file 3** Detailed sample information

**Additional file 4** Full fgsea analysis results

**Additional file 5** Full t-test results of (1) and (5)

## Data Availability

Raw and processed read data have been deposited into GEO under accessions GSE64810 and GSE129473. The GTEx data used in this manuscript were obtained from dbGaP under accession number phs000424 on 10/11/2017 (the data release at time of access was v7.p2, accessible under the ‘Study version history’ link of the current accession). All analysis and figure code for this project are available at bitbucket.org/bubfnexus/asymptomatic_hd_mrnaseq .

## Abbreviations

BA9: Brodmann area 9
CAU: Caudate nucleus
GSEA: Gene set enrichment analysis
HD: Huntington’s disease
