# Supplementary material for: The caudate nucleus undergoes dramatic and unique transcriptional changes in human prodromal Huntington’s disease brain
Source: BMC Med Genomics. 2019 Oct 16;12:137. doi: 10.1186/s12920-019-0581-9 (PMC6796419; doi:10.1186/s12920-019-0581-9)
Supplement: Supplementary file 2 — Additional file 2 PCA Outlier Analysis [file 12920_2019_581_MOESM2_ESM.pdf]

Additional File 2 - PCA of gene counts

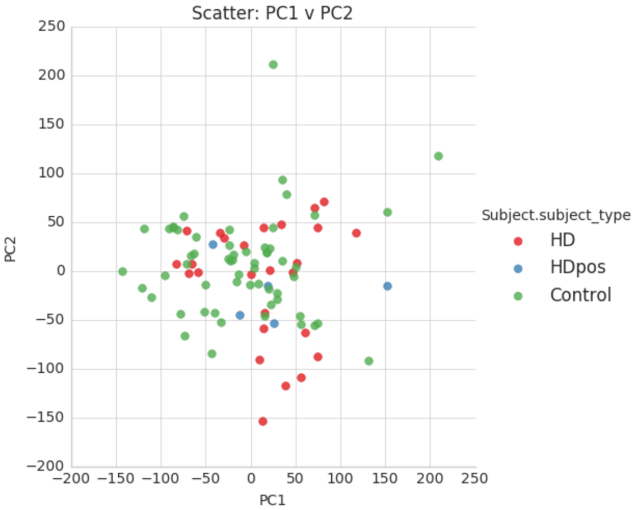

Figure 1. PCA identified no outliers that exerted a strong influence on the results in the first two components.

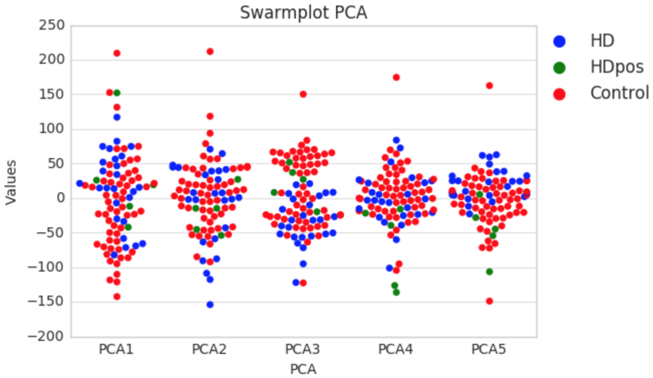

Figure 2. PCA swarm plot of the first five components by % explained variance. No strong outliers were removed based on these projections.
